# Supplementary material for: Maize Streak Virus: Single and Gemini Capsid Architecture
Source: Viruses. 2024 Nov 29;16(12):1861. doi: 10.3390/v16121861 (PMC11680415; doi:10.3390/v16121861)
Supplement: Supplementary file 1 [file viruses-16-01861-s001.zip › viruses-3266641-supplementary.pdf]

Maize Streak Virus: Single and Gemini Capsid Architecture

Antonette Bennett[1,†*], Joshua A Hull[1,†], Mario Mietzsch[1,†], Nilakshee Bhattacharya[2], Paul Chipman[1], Robert McKenna[1,*]

[1] Affiliation 1; Department of Biochemistry and Molecular Biology, College of Medicine Center for structural biology, Matt Knight Brain Institute, University of Florida, Fl 32610 - 0245, USA; dendena@ufl.edu (A.B.); mario.mietzsch@ufl.edu (M.M.); Joshua.hull@duke.edu (J.H.); pchipman@ufl.edu (P.C.); rmckenna@ufl.edu (R.M.)

[2] Affiliation 2; Biological Science Imaging Facility (BSIR), Department of Biology, 89 Chieftain Way Florida State University, USA; Nilakshee.bhattacharya@duke.edu (N.B.)

† These authors equally contributed to the work

* Correspondence: RM, phone: (352) 294-8396
AB, phone: (352) 294-8396

Supplemental Figures and Tables

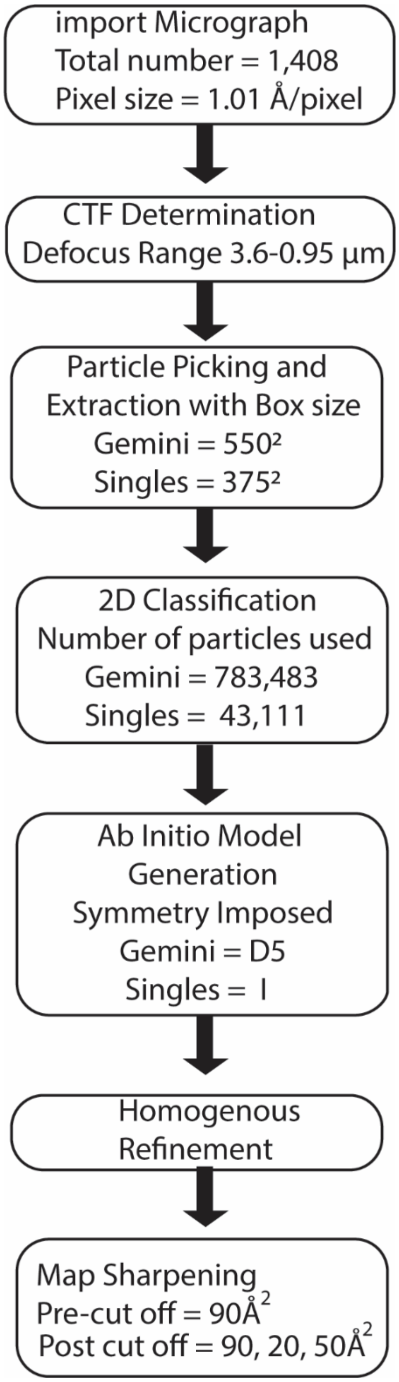

Figure S1 Cryo-EM workflow for the determination of theMSV-N [A] gemini and single capsid structure. The suite of subroutines were launched from the program cisTEM.

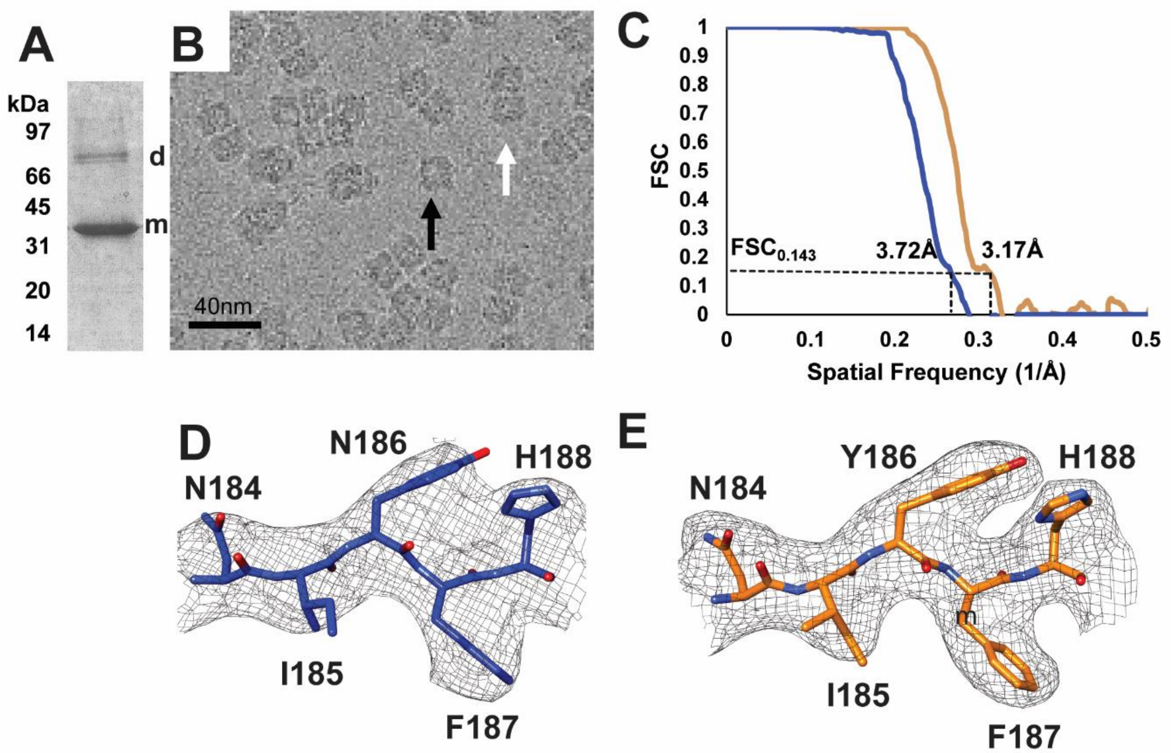

Figure S2 Cryo-EM of MSV-A [NG1] gemini and single capsid. (A) SDS PAGE of purified denatured sample showing CP monomer and dimer. (B) Vitrified micrograph of gemini (white arrow) and single (black arrow) capsid. (C) FSC plot of the gemini (orange) and single (blue) capsid map with $FSC_{0.143}$ = 3.17Å and 3.72Å respectively. (D) Electron density map (colored gray) and fitted model of MSV single (colored blue) showing aa184-188. (E) Electron density map (colored gray) and fitted model of MSV gemini showing aa184-188 (colored orange).

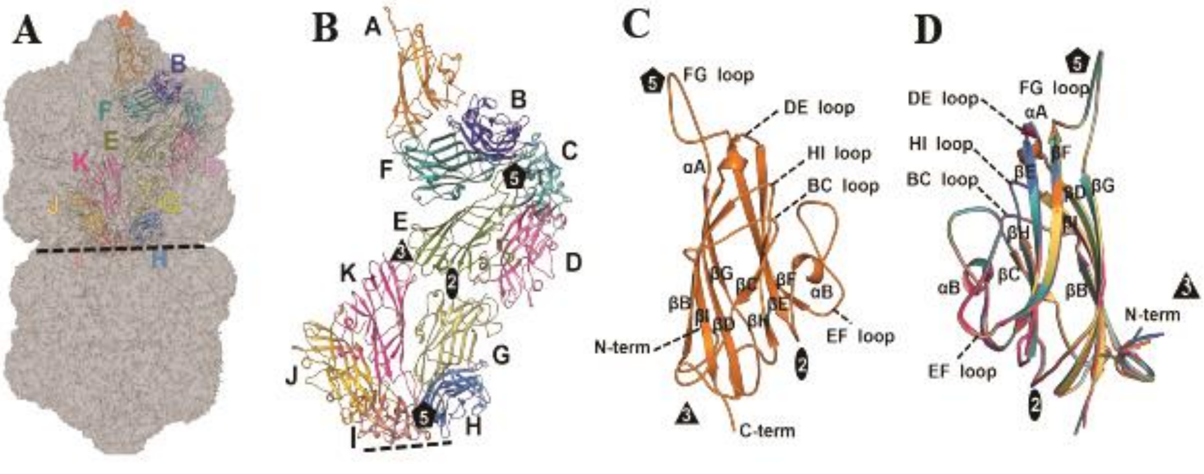

Figure S3 MSV-A [NG1] gemini structure and fitted model**.** (A) Surface representation colored gray and fitted viral asymmetric unit. B) Cartoon representation of the viral asymmetric unit labelled at the 2-, 3- and 5-fold axis. (C) Cartoon representation of the fitted monomer, which is colored orange, the secondary structural elements are labeled βBIDG, βCHEF, αA, and αB. (D) Superposition of the chain A, C, I and H.

The icosahedral 2-fold, 3-fold and 5-fold axes are represented as an oblong, triangle, and a pentagon, respectively.

Table S1 MSV-N[A] Nucleotide Binding Residues

Code: Nucleotide: #	Interaction	MSV
Y1: Cyt: 900	-	-
R2: Gua: 901	Hydrogen bond with $(PO_4)^{2-}$backbone, D^4base	R155
R3: Ade: 902	$(PO_4)^{2-}$backbone	R156
R3: Ade: 903	$(PO_4)^{2-}$backbone	K142
R4: Ade: 903	$(PO_4)^{2-}$backbone	R156
R4: Ade: 903	π stack	F190
Y5: Cyt: 904*	$(PO_4)^{2-}$backbone	R235
Y5: Cyt: 904*	C2′ of deoxyribose sugar	I42
Y6: Cyt: 905*	$(PO_4)^{2-}$backbone	R145, H149
Y7: Cyt: 906*	$(PO_4)^{2-}$backbone	H149
Y8: Cyt: 907	-	-
R9: Ade: 908	C2′ of deoxyribose sugar	K34

* and underline represent nucleotides that are π stacking with each other
- no interaction within 3.5Å distance

Table S2 Geminivirus Symmetry Related Interactions

Geminivirus	Symmetry	2-fold	3-fold	5-fold
**MSV - Single PDB ID: 8UH4**	**Chains**	**B:d**	**A:d**	**A:B**
	Buried surface area $(Å)^2$	267.6	271.1	1114.3
	No. of hydrogen bonds and salt bridges	2	3	6
**MSV - Gemini PDB ID: 8UGQ**	**Chains**	**A:B**	**A:F**	**B:F**
	Buried surface area $(Å)^2$	281	234.7	1666
	No. of hydrogen bonds and salt bridges	2	3	10
**ACMV - Gemini PDB ID: 6EK5**	**Chains**	**A:K**	**A:O**	**K:O**
	Buried surface area $(Å)^2$	437	74.9	920.8
	No. of hydrogen bonds and salt bridges	10	1	14
**AYVV - Gemini PDB ID: 6F2S**	**Chains**	**A:B**	**A:F**	**B:F**
	Buried surface area $(Å)^2$	605.3	335	949
	No. of hydrogen bonds and salt bridges	16	1	15

Table S3. Symmetry related Interactions of MSV Single and Gemini Capsid.

		(a)			
**MSV-N[A]**	**Symmetry**	**2-fold**	**3-fold**	**5-fold**	
**Singles**	**Chains**	**B:d**	**A:d**	**A:B**	
	Buried Surface area (Å)2	267.6	271.1	1114.3	
	Hydrogen bonds and salt bridges	Q212:C148	S71:R69, E73:N202	A139:Y186, V143:R235, D164:S177, R166:P173, S174, Y186:R156	
	Other bonds	H149:Q212, Q199	0	H91:W137, I171:R100, Y101, W178:A175, A176, S177, D164, N184:T104, H231:W137, R235: K142, V143	
	Total number of interactions / Interface	3	2	17	
**Gemini Apical - Peripentonal**	**Chains**	**A:B**	**A:F**	**A:DA**	**B:F**
	Buried Surface area (Å)^2	275.7	262.3	1557.6	1662
	Hydrogen bonds and salt bridges	Q212:E146, C148, H149, H149:Q199	R69:D72, R69:S71, D72, N243: E73	D89:K142, R156:Y186, E162:K179, R166:P173, A176, W178, R183:S102, Q233:T140, K142:Q233,	S102:R183, T140:Q233, R156:Y186, D164:W178, R166:S174, A176,
	Other bonds	0	N202:E73	T104:N184, W137:H91, D164:W178, W178:S177, C181:K179	H91:W137, I171:Y101, R166:P173, N175:W178, A176:R166, S177:D164, W178, K179:E162, K179:C181, N184:T104, Q233:K142
	Total number of interactions / Interface	4	5	15	17
			(b)		
**MSV-N[A]**	**Symmetry**	**2-fold**	**3-fold**	**5-fold**	**5-fold**

	Chains	E:G	E:K	E:D	G:K
**Gemini Peripentonal - Equatorial**	Buried surface area (Å)2	299.7	249.5	1658.8	1532
	Hydrogen bonds and salt bridges	Q212:C148, E146	E73:N202	E73:R145, S102:R183, T140:Q233, R156:Y186, R166:P173, A176	W137:H231, T140:Q233, R156:Y186, D164:W178, R166:P173, A176
	Other bonds	H149:Q212, Q199	0	Y101:I171, T104:N184, P131:Q46, T133:G49, W137:H91, D164:S177, W178, W178:S177, C181:K179	R100:I171, Y101:S169, T104:N184, T133:G49, W137:H91, K142:D89, K142:Q233, R145:I42, E162:K179, W178:N175, W178:S177, C181:K179
	Total number of interactions / Interface	4	1	15	18
**Gemini Equatorial - Equatorial (Dihedral)**	**Chains**	**GI:I**	**GI:H**	**I:H**	**GI:GH**
	Buried surface area (Å)^2	455.9	141.8	1454.3	1454.3
	Hydrogen bonds and salt bridges	Y130:D132, D132:K135,	S71:V203, D72:G119, Q120:E73	R166:P173, S174, A176, W178, R183:S102, Q233:T140	R166:P173, S174, A176, W178, R183:S102, Q233:T140
	Other bonds	E146:Q120 A121 Q125:Q125, D132,	0	T133:A48, G49, W137:H91, H231, I171:R100, N175:P173, W178, A176:W178, S177:D164,W178, K179:E162, C181, W184:T104, Q233:T140	T133:A48, G49, W137:H91, H231, I171:R100, N175:P173, W178, A176:W178, S177: D164, W178, K179:E162, C181, W184:T104, Q233:T140
	Total number of interactions / Interface	6	3	20	20

Table S4 Geminivirus sequence and structure comparison

		TPCTV	MSV	ACMV	AYVV	CCDAV	
% CP Sequence Identity	TPCTV	100	76	68	76	75	% Structural Similarity
	MSV	27	100	60	70	68	
	ACMV	25	23	100	85	68	
	AYVV	26	21	73	100	95	
	CCDAV	22	21	27	30	100	

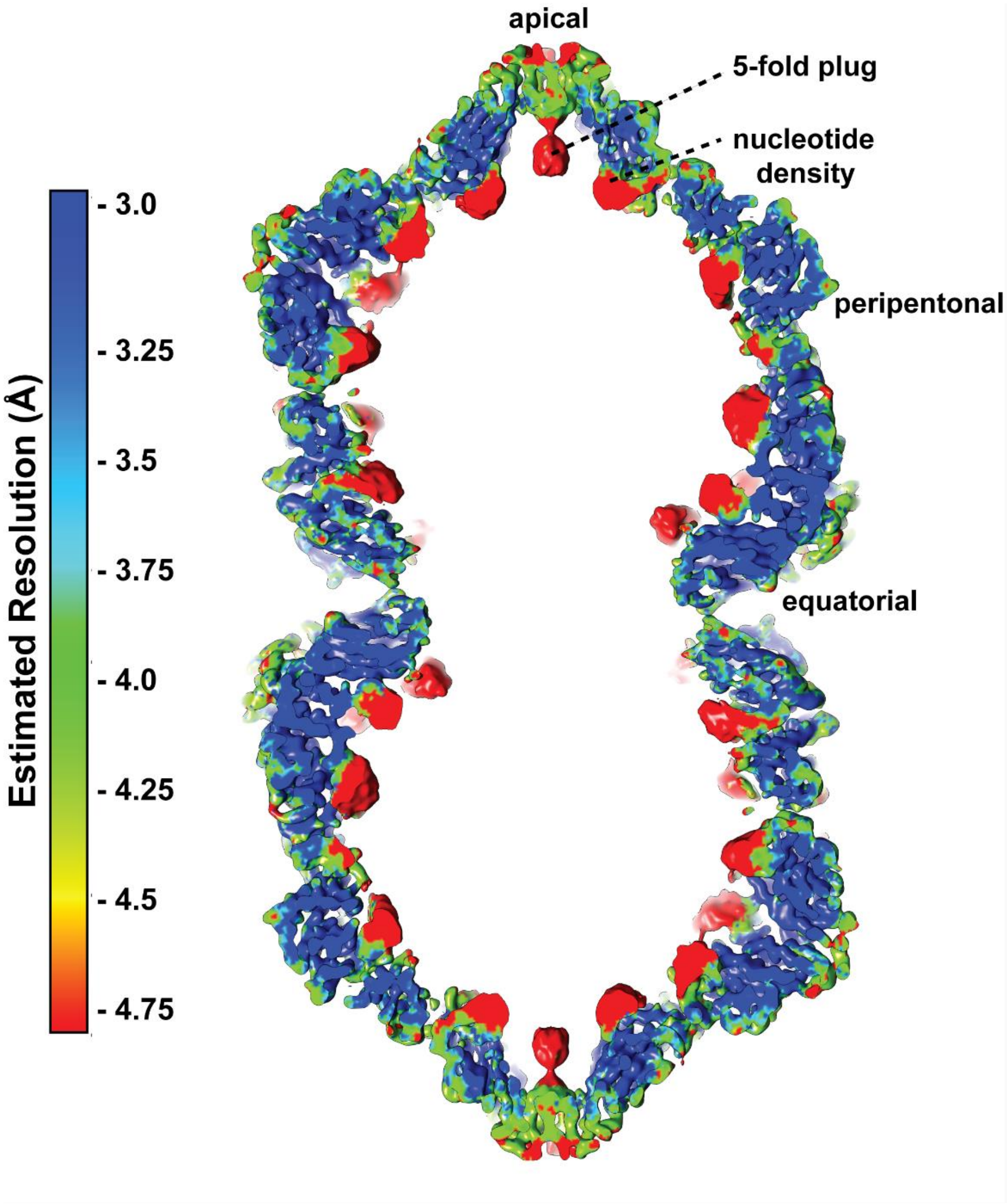

Figure S4 Local resolution of the cryo-EM gemini virus map determined by Resmap. Resolution key shown on the left with resolution range extending from 3.0Å – 4.75Å and colored blue – red, respectively.
